# Novel insights into the SLC7A11-mediated ferroptosis signaling pathways in preeclampsia patients: identifying pannexin 1 and toll-like receptor 4 as innovative prospective diagnostic biomarkers

**DOI:** 10.1007/s10815-022-02443-x

**Published:** 2022-03-24

**Authors:** Sarah Ragab Abd El-Khalik, Rowida Raafat Ibrahim, Muhammad Tarek Abdel Ghafar, Doaa Shatat, Omnia Safwat El-Deeb

**Affiliations:** 1 Medical Biochemistry & Molecular Biology Department, Faculty of Medicine, Tanta University, El Geesh Street, Tanta, 31511 Egypt; 2 Clinical Pathology Department, Faculty of Medicine, Tanta University, Tanta, Egypt; 3 Department of Gynecology and Obstetrics, Faculty of Medicine, Tanta University, Tanta, Egypt

**Keywords:** Preeclampsia, Ferroptosis, Oxidative stress, Pannexin 1, Toll-like receptor 4, Solute carrier family 7 member 11

## Abstract

**Purpose:**

Ferroptosis is associated with oxidative stress (OS) and is caused by iron-dependent lipid-peroxidative damage, but its role in PE is unclear. The aim of this study is to determine whether pannexin 1 (Panx1) and toll-like receptor 4 (TLR4) are key regulators of ferroptosis in PE.

**Methods:**

The study included 65 patients with PE and 25 healthy pregnant women. In normal and PE placental tissues, OS and ferroptosis markers, including Fe^2+^, malondialdehyde (MDA), reduced glutathione (GSH) levels, heme oxygenase-1 (HO-1) and glutathione peroxidase 4 (Gpx4) activity, were estimated. Panx1 and solute carrier family 7 member 11 (SLC7A11) mRNA expression levels were relatively quantified in placental tissues using real‐time polymerase chain reaction (RT‐PCR), while serum Panx1, serum TLR4, and placental activating transcription factor 3 (ATF3) levels were measured by ELISA.

**Results:**

In placental tissues, Panx1 and TLR4 expression levels were significantly increased in patients with PE compared to controls and were positively correlated with pro-ferroptosis mediators such as placental Fe^2+^ and MDA levels and negatively correlated with anti-ferroptosis regulators such as placental GSH level, HO-1, and Gpx4 activity. Additionally, Panx1 and TLR4 had a positive correlation with ATF3 and a negative correlation with SLC7A11. Serum Panx1 and TLR4 levels were positively correlated with their placental tissue expression and showed good diagnostic capabilities for ferroptosis in PE.

**Conclusion:**

Therefore, Panx1 and TLR4 are suggested to induce ferroptosis in PE via SLC7A11-mediated signaling pathways, offering a novel perspective on PE pathogenesis and novel diagnostic tools for PE.

## Introduction

Preeclampsia (PE) is a life-threatening pregnancy disorder with a global prevalence of approximately 5–7% and a considerable maternal and perinatal mortality rate [1]. It is widely accepted that the pathogenesis of PE is associated with incomplete spiral artery transformation, resulting in hypoperfusion and placental ischemia, which triggers pro-inflammatory factors during aberrant placentation. It also promotes the release of reactive oxygen species from the cell membrane, endoplasmic reticulum, and mitochondria, which causes protein and DNA damage [2, 3]. Within the placental villous trophoblast, dysregulation of cell biological processes is associated with several types of programmed cell death that have been suggested to be a crucial mechanism in the pathogenesis of PE. Enhanced apoptotic events are involved in abnormal placentation associated with impaired invasion, vascular remodeling, and microcirculation in the placenta [4].

Ferroptosis was initially identified as a regulated form of iron-dependent non-apoptotic cell death that is distinct from apoptosis and necrosis morphologically, genetically, and biochemically [5]. Ferroptosis is characterized by increased ferrous iron (Fe^2+^) transport in cells, which results in reduced glutathione (GSH) depletion and glutathione peroxidase 4 (Gpx4) inactivation. When free iron is present, Gpx4 catalyzes the reduction of lipid hydroperoxides to non-reactive lipid alcohols; lipid hydroperoxides can participate in Fenton’s reactions [6]. Furthermore, solute carrier family 7 member 11 (SLC7A11) is a cystine/glutamate antiporter that imports cystine into cells while exporting glutamate, promoting GSH biosynthesis, and protecting cells from oxidative stress (OS) [7]. Although dysregulated ferroptosis has been implicated in a variety of pathological conditions and metabolic pathways, including those involving iron, polyunsaturated fatty acids, NADPH oxidase, heme oxygenase-1 (HO-1), and coenzyme Q10 [8, 9], the exact molecular mechanisms underlying ferroptosis in PE remain unknown.

Pannexin 1 (Panx1) is a transmembrane channel protein that connects the intracellular and extracellular spaces. Thus, it allows the passage of ions and small molecules. It is expressed in a wide variety of human tissues, including the brain, heart, lung, placenta, blood endothelium, and erythrocytes [10]. Panx1 has been implicated in the basic model of purinergic signaling nucleotide release and in the regulation of a variety of physiological and pathophysiological processes, including apoptosis, pyroptosis, tumor cell metastasis, and vasoconstriction [11]. Indeed, it has been implicated in ischemia-induced seizures, strokes, hypertension, inflammation, and neuropathic pain. Panx1 interaction with a diverse array of signaling molecules mediates key cellular processes [12, 13]. Despite widespread interest, little is known about Panx1’s role in PE, and the relationship between Panx1 and ferroptosis remains unclear.

Toll-like receptors (TLRs), which are important components of innate immunity, are expressed in normal and pre-eclamptic placentas as well as decidual cells [14]. Among the members of the TLR family, TLR4 is expressed in extravillous trophoblasts, including endovascular and interstitial trophoblasts in the placenta. It is activated by damage-associated molecular patterns induced by increased OS and promotes nuclear factor-kappa B (NF-ĸB) activation, cytokine synthesis, TLR-triggered inflammatory responses, and placental cytotrophoblast apoptosis. Thus, altered TLR4 expression and function at the maternal–fetal interface play a crucial role in the development of PE by establishing a link between OS and a locally altered cytokine environment [15]. Furthermore, it was found that silencing TLR4 suppresses autophagy and ferroptosis [16]. To date, the significance of TLR4 and its relationship to ferroptosis in the context of PE remains unclear. So, this study aims to focus on both Panx1 and TLR4, which act as key regulators of ferroptosis, and to explore their molecular mechanisms in the pathogenesis of PE.

## Subjects and methods

### Study population

This study included 90 pregnant women in their third trimester (28 to 38 weeks of gestation) and aged 20 to 45 years old who were admitted to the Obstetrics and Gynecology department at Tanta University Hospitals between January 2020 and April 2021. They were divided into two groups: the first included 25 healthy pregnant women with normal blood pressure as a control group, while the second included 65 patients with PE. According to international classification systems [17], PE was defined as a persistent increase in blood pressure after 20 weeks of gestation, with a systolic blood pressure (SBP) ≥ 140 mmHg and/or a diastolic blood pressure (DBP) ≥ 90 mmHg, accompanied by proteinuria (24-h urinary protein ˃ 300 mg, or proteins ˃ + 1 in a random urine dipstick test, or random urinary proteins ˃ 30 mg/dL). Blood pressure should be elevated on at least two occasions, 6 h apart. To minimize potential confounding factors, women with chronic hypertension, gestational or pre-gestational diabetes, and chronic renal or hepatic diseases and those taking long-term steroid, immunosuppressive, or cytotoxic therapy were excluded from this study.

All study participants had their body weight, height, and blood pressure measured and their body mass index (BMI) calculated. Lower limb edema was examined clinically, and proteinuria was detected using a urine dipstick test in random urine samples. Additional data were collected on pre-pregnancy weight, reproductive and medical history, and delivery, including gestational age, mode of delivery, antenatal steroid therapy, and labor induction.

The study protocol was approved by the local research ethics committee of the Faculty of Medicine at Tanta University and adhered to the principles of the Declaration of Helsinki II. Prior to enrollment in this study, all participants gave written informed consent. The sample size was calculated using Open Epi software, with a confidence level of 95% and a power of 80%. According to a previous study, mean TLR4 level in patients with PE was 3.76 ± 1.07 ng/ml compared to 2.43 ± 1.69 ng/ml in healthy controls [18]. As a result, the adequate sample size is calculated to be 50, with 25 in each group.

### Methods

#### Blood sampling

Blood samples were collected from all participants under strict sterile conditions immediately after delivery, gently delivered into sterile plain vacutainers, allowed to clot at room temperature, and then centrifuged at 1200 g for 10 min. Sera were collected to measure Panx1 and TLR4 levels. Samples were stored frozen at − 80 °C until analysis.

#### Placental tissue sampling:

The placental tissues were collected immediately after delivery, dissected, weighted, and fractionated into three parts. The first part was frozen at − 80 °C for quantitative real-time polymerase chain reaction (RT-PCR) analysis of Panx1, TLR4, and SLC7A11 mRNA expression. Microsomal separation was performed on the second part to determine HO-1 activity. The third part was homogenized 1:10 (w/v) in phosphate buffer saline (PBS) at pH 7.4 with a homogenizer, centrifuged at 10,000 rpm for 15 min, and the supernatant was separated and used to estimate activating transcription factor 3 (ATF3), malondialdehyde (MDA), GSH, and Fe^2+^ levels, as well as Gpx4 activity.

#### Biochemical assays

##### Determination of placental Fe^2+^

The placental Fe^2+^ level was measured calorimetrically using a commercial iron assay kit (Abcam, Japan, Catalog No. ab83366) according to the manufacturer’s instructions. Ten milligrams of placental tissues was washed in cold PBS, homogenized in iron assay buffer, centrifuged, and the supernatant was used for the assay. The samples and standards were applied to a 96-well microtiter plate, and 5 μl of iron reducer or buffer solution was added to each standard or sample, respectively. The plate was then mixed and incubated for 30 min at 37 °C. After adding 100 μl of the iron probe to each well, mixing and incubating at 37 °C for 60 min, the absorbance was read at a wavelength of 593 nm on a microplate reader.

##### Determination of placental MDA level

Lipid peroxidation was detected as previously described by Ohkawa et al. [19]. In a 10-ml centrifuge tube, 250 μl homogenate was shaken with 1.25 ml of trichloroacetic acid (20%). Then, 0.5 ml of thiobarbituric acid was added, shaken, and heated for 30 min in a boiling water bath, followed by rapid cooling. After adding 2.0 ml of N-butanol, the tubes were shaken and centrifuged at 3000 rpm for 10 min. The subsequent N-butanol layer was separated, and the absorbance was read at a wavelength of 532 nm against a blank. The results are expressed in nmol/ml.

##### Determination of placental Gpx-4 activity

The Gpx-4 activity was measured using a colorimetric commercial kit purchased from Biodiagnostic Co., Giza, Egypt, as described by Paglia et al. [20], in which 0.01 ml of sample was added to 1 ml of assay buffer, 0.1 ml of NADPH reagent, and 0.1 ml of the substrate (H_2_O_2_) and gently mixed. The decrease in absorbance was measured over a 3-min period against deionized water.

##### Determination of placental reduced GSH level

Reduced GSH levels in placental homogenate were measured using a commercial kit (Biodiagnostic, Egypt), as previously described by Beutler et al. [21]. The method is based on the reduction of 5, 5′-dithiobis-2-nitrobenzoic acid (DTNB) by GSH, which results in a yellow compound that is measured as absorbance at 405 nm. The absorbance is directly proportional to the GSH concentration in mg/dl.

##### Determination of placental HO-1 activity

Placental microsomes were isolated using the Schenkman and Cinti method [22]. They were then used to measure microsomal HO activity, which was done according to the modified Tenhunen et al. method [23].

##### Determination of serum Panx1, serumTLR4, and placental ATF3

All were measured using a specific enzyme-linked immunosorbent assay (ELISA) kit (MyBiosource, Inc., San Diego, CA, Catalog No. MBS764744, MBS765181, and MBS009530, respectively). The intensity of the resulting colors was measured at 450 nm on a Tecan Spectra II microplate reader (Hombrechtikon, Switzerland), and the results of each sample were obtained from the standard curve.

##### Quantitative RT-PCR analysis of Panx1, TLR4, and SLC7A11

Total RNA was isolated from placental tissues using the GeneJET RNA purification kit (Thermo Fisher Scientific, Waltham, Massachusetts, USA) according to the manufacturer’s protocol. RNA concentration and purity were determined using a NanoDrop spectrophotometer (NanoDrop Technologies, Inc., Wilmington) to measure optical density at 260 nm and the ratio 260/280 nm, respectively, and then the RNA was stored at − 80 °C. Total RNA was reverse transcribed using RevertAid H Minus reverse transcriptase (Thermo Fisher Scientific) Waltham, Massachusetts, USA) to generate cDNA. The cDNA was then stored at − 20 °C until use. cDNA was used as a template to detect the relative expression of Panx1, TLR4, and SLC7A11 using the StepOnePlus real-time PCR system (Applied Biosystem, Foster City, CA). GAPDH was used as a reference gene. Table 1 shows the primer sequences used in this analysis. cDNA was amplified using the following thermal profile: initial denaturation at 95 °C for 10 min, 40 cycles (denaturation for 15 s at 95 °C, annealing for 30 s at 60 °C, and extension for 30 s at 72 °C). The cycle threshold (Ct) values of the target genes and housekeeping gene were determined, and the relative gene expression was calculated using the 2^−ΔΔ*C*t^ method [24].

**Table 1:** Primer sequences of genes that are used in RT-qPCR assay

	Forward primer	Reverse primer	GenBank accession no
Panx1-1	5′ CTGTGGACAAGATGGTCACG 3′	3′CAGCAGGATGTAGGGGAAAA 5′	#NM_015368.4
TLR4	5′ AGCCACGCATTCACAGGG 3′	5′ CATGGCTGGGATCAGAGTCC 3′	#NM_003266.4
SLC7A11	5′ TCTCCAAAGGAGGTTACCTGC 3′	5′AGACTCCCCTCAGTAAAGTGAC 3′	#NM_014331.4
GAPDH	5′ ATGGGGAAGGTGAAGGTCG 3′	3′GAGGTCAATGAAGGGGTCAT 5′	#NM_002046.7

*Panx1* Pannexin 1, *SLC7A11* solute carrier family 7 member 11, *TLR4* toll-like receptor 4, *GAPDH* glyceraldehyde 3-phosphate dehydrogenase

### Statistical analysis

The statistical analyses were performed using the Statistical Package for the Social Sciences (SPSS) version 23 (Chicago, IL, USA). All data are assumed to be normally distributed and are expressed as mean and standard deviation (SD). The unpaired Student’s *t*-test was used to analyze the difference between the two studied groups. Categorical data were expressed as numbers and percentages and compared between the two studied groups using the chi-square *χ*^2^ test. The relationship between tissue ferroptosis mediators and PE was determined using binary logistic regression analysis adjusted for confounding factors such as gestational age. A Pearson’s correlation analysis was conducted to study the relationship between the different parameters. A receiver operator characteristic (ROC) curve was constructed to assess the performance characteristics of serum Panx1 and TLR4 with the area under the curve (AUC). The optimal cutoff values of these markers were determined via Youden’s index. A *p*-value of less than 0.05 was considered statistically significant.

## Results

### Demographic and clinical characteristics of the studied groups

This study included 65 patients diagnosed with PE and 25 healthy controls. Both groups were age-matched. The majority of patients with PE is multiparous and has a positive family history of PE. They also had bilateral lower limb edema and proteinuria greater than + 2. Meanwhile, patients with PE had significantly higher SBP and DBP and a shorter gestational age than the control group, with no significant difference in BMI between the two groups. With regard to the delivery data, no cases or controls were subjected to labor induction. The PE group had a significantly lower gestational age than the control group, but no significant difference in the mode of delivery or rate of antenatal steroid therapy was observed between the two groups (Table 2).

**Table 2:** Demographic and clinical characteristics of the studied groups

		Control group (*n* = 25)	PE group (*n* = 65)	*t*/*χ *^*2*^
Age (years)		28.52 ± 3.44	30.14 ± 4.14	1.735
Gestational age (weeks)		37.24 ± 1.90	35.62 ± 2.14	3.314*
BMI (kg/m^2^)		26.88 ± 2.4	28.05 ± 2.6	1.95
SBP (mmHg)		105.8 ± 7.314	165.1 ± 2.4	22.84 *
DBP (mmHg)		76.8 ± 5.93	106.8 ± 9.46	14.79 *
Parturitions *n* (%)	Primiparous *n* (%)	10 (40)	25 (38.46)	0.018 *
	Multiparous *n* (%)	15 (60)	40 (61.54)	
Proteinuria ˃ 2 + *n* (%)		0 (0)	42 (64.62)	
Family history of PE *n* (%)		0 (0)	54 (83.07)	
Bilateral lower limb edema *n* (%)		0 (0)	61 (93.85)	
Antenatal steroid therapy *n* (%)		4 (16)	9 (13.8)	0.068
Mode of delivery, CS *n* (%)		8 (32)	23 (35.4)	0.092

Data presented as means ± SD

*n* number, *t* unpaired student *t*-test, χ^2^ chi-square test, *PE* preeclampsia, *BMI* body mass index, *SBP* systolic blood pressure, *DBP* diastolic blood pressure, *CS* caesarean section

^*^Significant *P* value < 0.05

### Ferroptosis and oxido-inflammatory status in PE

Ferroptosis mediators were significantly altered in PE with increased placental Fe^+2^ levels (Table 3). Additionally, MDA levels, a biomarker of lipid peroxidation, were significantly increased while HO-1 activity, Gpx4 activity, and GSH levels were significantly decreased in the placental tissues of patients with PE compared to the control group (*P* < 0.05) (Table 3), indicating an imbalance between pro-oxidants and antioxidants in patients with PE favoring the development of an oxidative stress state.

**Table 3:** Placental tissue biomarkers of ferroptosis and oxidative stress of the studied groups

	Control group (*n* = 25)	PE group (*n* = 65)	Unpaired student *t* test (*t*)
Placental Fe^2+^ (nmol/mg tissue)	0.223 ± 0.49	0.439 ± 0.082	12.34*
Placental HO-1 activity (nmol bilirubin/mg tissue ptn/hour)	1.466 ± 0.2	0.702 ± 0.11	22.99*
Placental GSH (µg/mg tissue)	55.2 ± 11.35	24.13 ± 3.46	19.94*
Placental Gpx-4 activity (U/gm tissue)	6.79 ± 0.87	4.106 ± 0.62	16.28*
Placental MDA (nmol/mg tissue)	0.499 ± 0.12	0.98 ± 0.28	8.53*
Placental ATF3 (pg/gm tissue)	12.87 ± 1.94	23.36 ± 3.28	11.41*

Data presented as means ± SD

*Fe*^*2*+^ ferrous iron, *HO-1* heme oxygenase 1, *GSH* reduced glutathione, *Gpx-4* glutathione peroxidase 4, *MDA* malondialdehyde, *ATF3* activating transcription factor 3, *t* unpaired student *t*-test

^*^Significant *P* value < 0.05

### Panx1 and TLR4 mRNA expression and ATF3 levels in PE

**Fig. 1:**
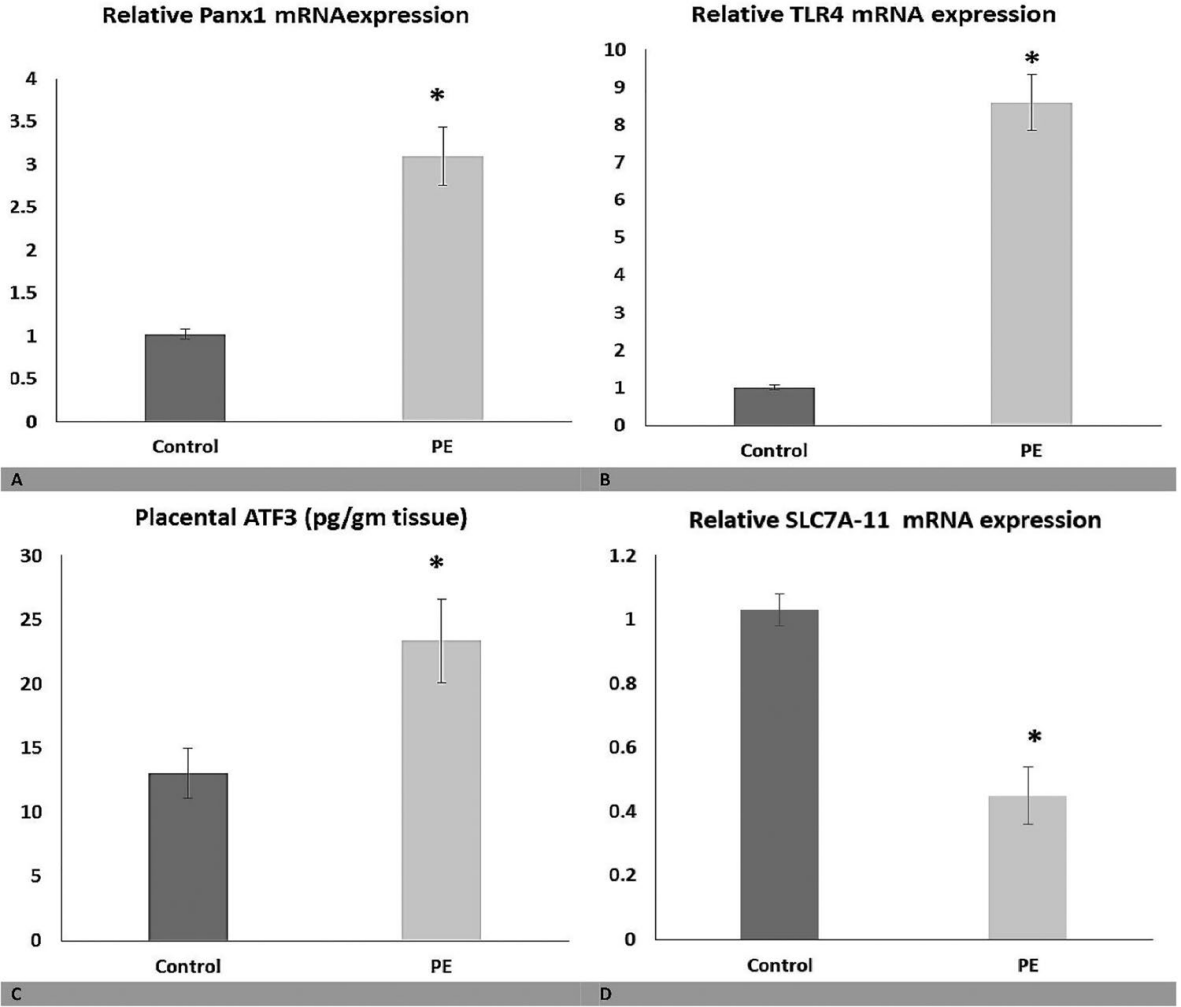
Relative placental mRNA Panx1, SLC7A11, and TLR4 mRNA gene expression and placental ATF3 level in both studied groups. Data are expressed as Mean ± SD. *P* was considered significant at ≤ 0.05*

Panx1 (Fig. 1a) and TLR4 mRNA expression (Fig. 1b) were significantly increased in the placental tissues of patients with PE compared to the control group, adjusted to the gestational age (odds ratio (OR): 361.6, 95% confidence interval (CI): 1.992–6566.0, *P* = 0.026; OR: 3.932, 95% CI: 1.215–12.726, *P* = 0.022, respectively). As a result, Panx1 and TLR4 have been linked to PE. Both Panx1 and TLR4 expression levels in the placental tissues were positively correlated with each other and with markers of ferroptosis such as placental Fe^+2^ (*P* < 0.05) and OS such as MDA, but were negatively correlated with antioxidants such as placental HO-1 activity, GSH, and Gpx-4 (*P* < 0.05) (Table 4). Thus, Panx1 and TLR4 are linked to ferroptosis and OS in PE.

**Table 4:** Pearson’s correlation of placental Panx1, TLR4, SLC7A11, and ATF3 with other studied parameters in PE group

	Placental Panx1		Placental TLR4		Placental SLC7A11		Placental ATF3	
	*r*	*P* value	*r*	*P* value	*r*	*P* value	*r*	*P* value
Placental Fe^+2^ (nmol/mg tissue)	0.550	< 0.001*	0.556	< 0.001*	− 0.549	< 0.001*	0.232	0.036*
Placental HO-1 activity (nmol bilirubin/ mg tissue ptn/hour)	− 0.500	< 0.001*	− 0.408	< 0.001*	0.463	< 0.001*	− 0.249	0.041*
Placental MDA (nmol/mg tissue)	0.209	0.09	0.306	0.013*	− 0.366	0.002*	0.069	0.587
Placental GSH (µg/mg tissue)	− 0.624	< 0.001*	− 0.562	< 0.001*	0.529	< 0.001*	− 0.107	0.396
Placental Gpx activity (U/gm tissue)	− 0.685	< 0.001*	− 0.545	< 0.001*	0.622	< 0.001*	− 0.080	0.527
Placental Panx1	–	–	0.764	< 0.001*	− 0.769	< 0.001*	0.258	0.038*
Placental TLR4	0.764	< 0.001*	–	–	− 0.887	< 0.001*	0.153	0.025*
Placental SLC7A11	− 0.769	< 0.001*	− 0.887	< 0.001*	–	–	− 0.165	0.189
Placental ATF3 (pg/gm tissue)	0.258	0.038*	0.153	0.025*	− 0.165	0.189	–	–

*Fe*^+*2*^ ferrous iron, *HO-1* heme oxygenase 1, *GSH* reduced glutathione, *Gpx-4* glutathione peroxidase 4, *MDA* malondialdehyde, *Panx1* pannexin 1, *TLR4* toll-like receptor 4, *SLC7A11* solute carrier family 7 member 11, *ATF3* activating transcription factor 3

^*^Significant *P* value ≤ 0.05

ATF3 levels were significantly increased in the placental tissues of patients with PE, adjusted to gestational age (OR: 4.995, 95% CI: 1.439–17.340, *P* = 0.011) (Fig. 1c) and were positively correlated with pro-ferroptosis mediators, placental Fe^+2^, and negatively correlated with anti-ferroptosis mediators, placental HO-1 activity (*P* < 0.05) (Table 4). Thus, elevated ATF3 levels are linked to ferroptosis in PE.

SLC7A11 mRNA expression was significantly downregulated in placental tissues of patients with PE compared to healthy controls, adjusted to gestational age (OR: 0.877, 95% CI: 0.828–0.929, *P* < 0.001) (Fig. 1d) and was negatively correlated with pro-ferroptosis mediators such as placental Fe^+2^ and MDA, but positively correlated with anti-ferroptosis mediators such as placental HO-1 activity, GSH, and Gpx-4 (*P* < 0.05) (Table 4). Thus, downregulated SLC7A11 expression is linked to ferroptosis in PE.

In addition, placental Panx1 and TLR4 were negatively correlated with placental SLC7A11 and positively correlated with placental ATF3, indicating a significant interaction in favor of ferroptosis (Table 4).

The placental tissue expression of ferroptosis mediators was assessed in the PE group according to the mode of delivery and antenatal steroid therapy and revealed no significant difference in placental Panx1, TLR4, and SLC711A expression and ATF3 levels between those delivered normally and those delivered via elective cesarean section, as well as between those who received antenatal steroid therapy and those who did not (all *P* > 0.05).

### Diagnostic accuracy of Panx1 and TLR4 for ferroptosis in PE

**Fig. 2:**
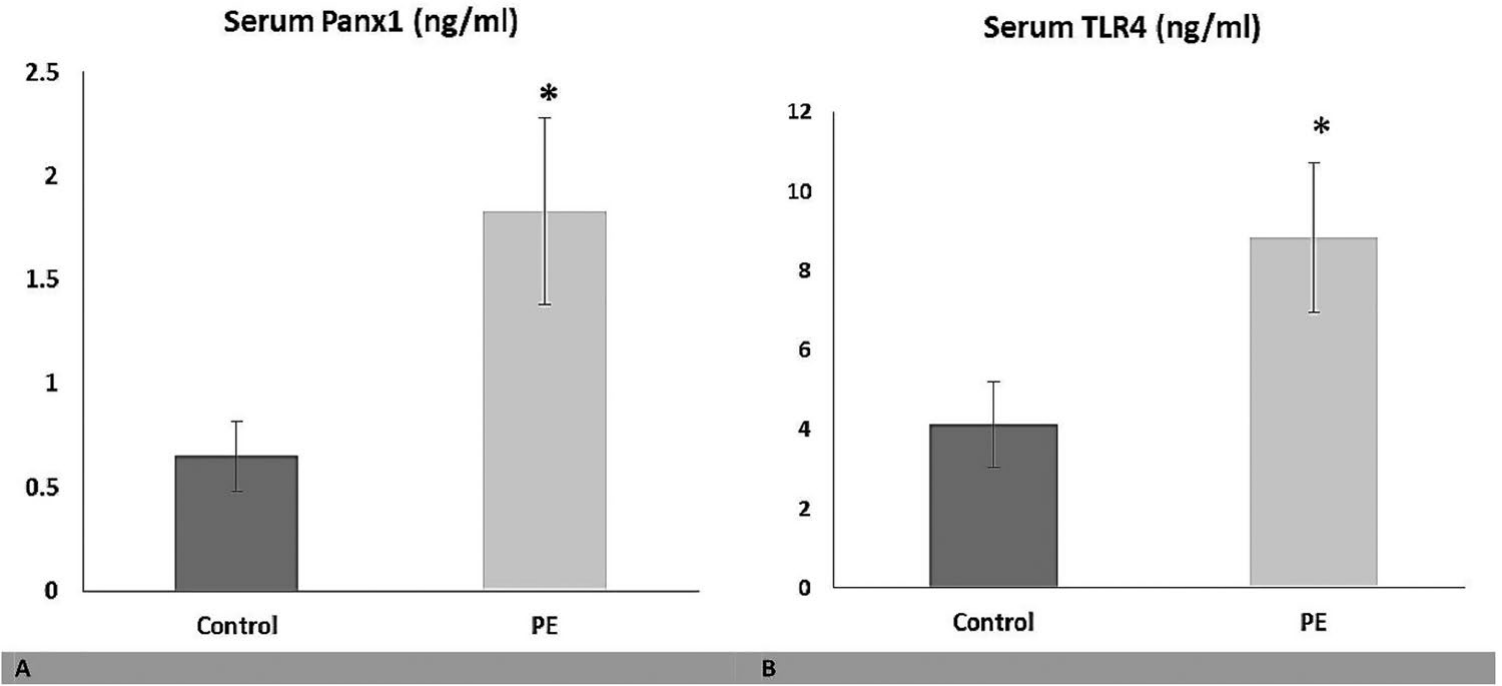
Serum levels of **A** Panx1 and **B** TLR4 in both studied groups. Data are expressed as Mean ± SD. *P* was considered significant at ≤ 0.05*

**Fig. 3:**
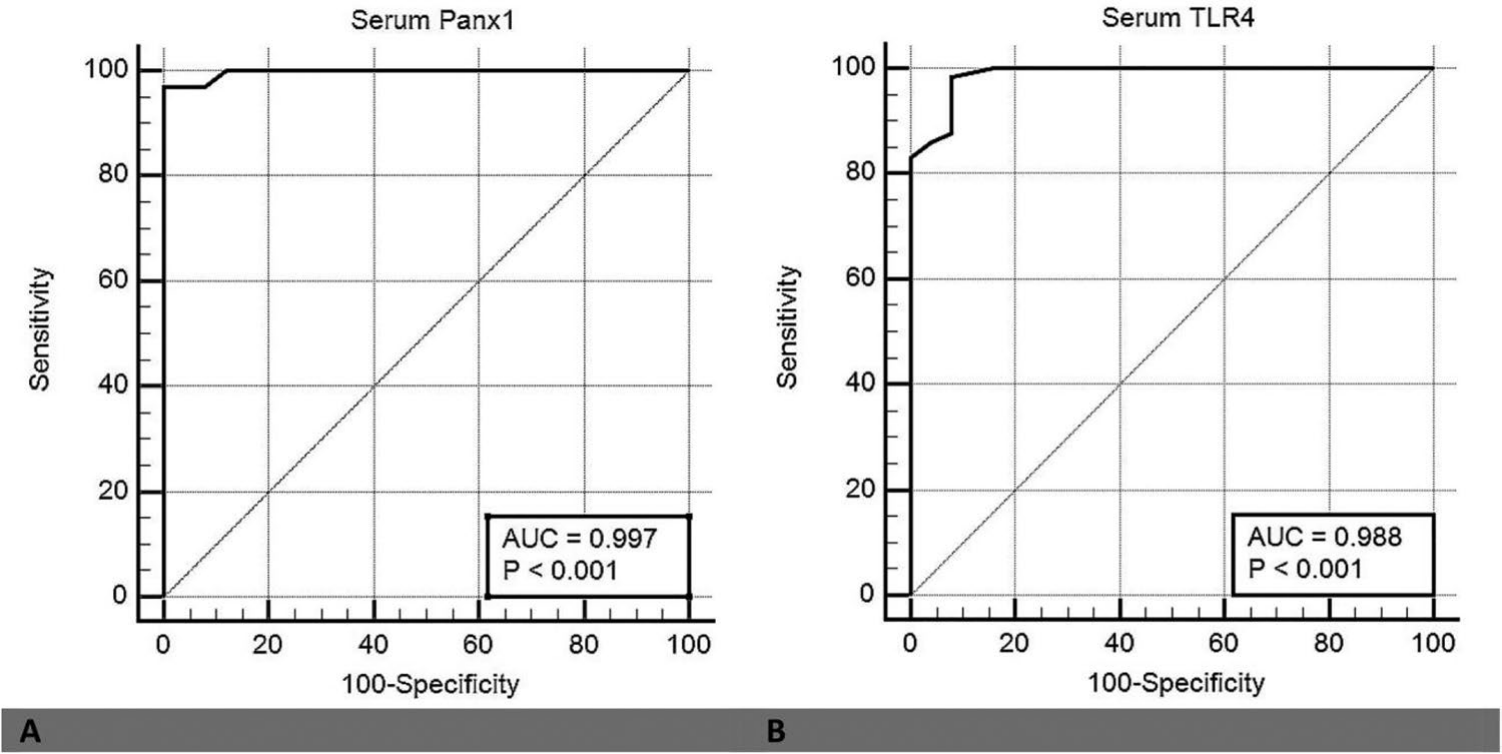
Receiving operating characteristic (ROC) curve for **A** serum Panx1 and **B** serum TLR4 as diagnostic tools for ferroptosis in PE patients

The serum Panx1 and TLR4 levels were significantly higher in patients with PE than in the control group (*P* < 0.05) (Fig. 2). The Pearson’s correlation analysis revealed a positive correlation between serum and placental Panx1 (*r* = 0.559, P < 0.01) as well as serum and placental TLR4 (*r* = 0.884, P < 0.01), indicating that serum Panx1 and TLR4 adequately reflect their placental expression and can be used as serological biomarkers for ferroptosis in PE. Thus, ROC curve analyses were used to assess the diagnostic performance of serum Panx1 and TLR4 in detecting placental affection in patients with PE (Fig. 3). Our data revealed that serum Panx1 has an AUC of 0.997 (95% confidence interval (CI): 0.954–1.000) with a sensitivity of 96.9% and a specificity of 100% at a cutoff value of > 0.94 ng/ml. Besides, at a cutoff value of > 5.30 ng/ml, serum TLR4 has a sensitivity of 98.5%, a specificity of 92%, and an AUC of 0.988 (95% CI: 0.938–1.000). There is no statistically significant difference in AUC between serum Panx1 and TLR4 (*P* = 0.339).

## Discussion

PE is thought to be caused by abnormal placentation, endothelial dysfunction, inflammation, and OS, among other causes [25]. Ferroptosis is a novel form of programmed cell death that is caused by iron-dependent lipid peroxidative damage [26].

Interestingly, labile Fe^2+^ is required for the intracellular accumulation of lipid peroxide molecules during ferroptosis. The current study revealed, for the first time, a significant increase in placental Fe^2+^ in patients with PE. This is in agreement with several studies reporting that total serum iron is higher in PE than in a healthy pregnancy, and that serum iron and blood pressure are directly correlated [27–29]. This excess iron can react with damaged placental cell membrane free radicals and circulating lipoproteins, causing lipid peroxidation in the placenta and vasculature [30]. Fe^2+^ also generates hydroxyl free radicals in the Fenton reaction, which induce inflammation and contribute to OS [31], mostly in trophoblast cells. This causes shallow endovascular invasion of extra-villous cytotrophoblast cells and suboptimal remodeling of the maternal spiral arteries, which are both pathologic hallmarks of PE [32].

During ferroptosis, iron-induced lipid peroxidation causes catastrophic membrane rupture, reducing Gpx4 activity. Among Gpx family members, Gpx4 is unique in its ability to reduce phospholipid hydroperoxide to lipid alcohols, preventing toxic lipid accumulation and maintaining cellular lipid homeostasis [33]. Thus, it is one of the major antioxidant systems that prevent the onset of an oxidative iron-dependent ferroptosis [34]. GSH, the primary cofactor for Gpx4 activity, is a type 1 ferroptosis stimulator when depleted [35]. In our study, Gpx4, GSH, and MDA levels were found to be significant ferroptosis-linked indicators in PE patients. Anti-ferroptosis factors, GSH and Gpx4, decreased significantly while the pro-ferroptosis factor, MDA, increased significantly in patients with PE compared to controls. These findings are consistent with previous reports [36–38].

TLR4, a pattern recognition receptor, is expressed in amniotic, intermediate, and extravillous trophoblast cells during normal pregnancy. The high serum TLR4 levels in patients with PE suggest its potential pathogenic role in PE pathogenesis and its use as a highly accurate biomarker for diagnosing PE, which is consistent with previous studies [39, 40]. TLR4 is an important regulator of apoptosis and autophagy [41], and its upregulation may be involved in ferroptosis [16]. At the same time, TLR4 inhibition reduces OS-induced damage, ferroptosis activation, and inflammation [42]. Although the link between PE and TLR4 activation is well known, the effects of TLR4 signal transduction pathways on PE in ferroptosis remain unclear. In this study, TLR4 correlated positively with pro-ferroptosis mediators and negatively with anti-ferroptosis mediators in patients with PE, indicating a role in ferroptosis. Also, the TLR4 level in serum correlates positively with its placental expression and has a high diagnostic power of 0.988 for ferroptosis in PE.

SLC7A11 (system Xc^−^) imports cystine for glutathione biosynthesis, and its loss reduces intracellular GSH levels. Thus, in PE under hypoxic conditions, GSH lack and intracellular Fe^2+^ accumulation are expected to occur during ferroptosis of trophoblasts [39]. Similarly, SLC7A11 expression is downregulated in placental tissues of patients with PE and correlates negatively with pro-ferroptosis mediators and positively with anti-ferroptosis mediators, indicating that its reduction promotes ferroptosis in PE.

The placenta has the highest levels of ATF3 mRNA expression compared to other human tissues. Thus, it may be effective in placental biology and pathology [43]. This matched our findings, where we found significantly higher levels of placental ATF3 in the PE group than in the control group. Previous studies have linked TLR4/NF-κB signaling to ATF3 expression [44]. By binding to the SLC7A11 promoter, ATF3 suppresses the system Xc^−^, predisposing cells to ferroptosis. Conversely, overexpressing SLC7A11 inhibits ATF3-mediated ferroptosis [45]. In the present study, TLR4 has a positive correlation with ATF3 and a negative correlation with SLC7A11, indicating for the first time that TLR4/ATF3/SLC7A11 signaling is a novel mechanism of ferroptosis in PE.

Antenatal corticosteroid therapy is effective in preventing preterm labor and adverse neonatal outcomes by promoting fetal lung maturation and neuroprotection [46]. Although dexamethasone has been shown to induce osteoporosis by promoting ferroptosis via enhancing lipid peroxidation and altering ferroptosis marker expression, resulting in the downregulation of GPX4 and system Xc^−^ in osteoblasts [47], the mechanisms by which it induces ferroptosis in PE have not been previously investigated. The current study revealed no significant difference in placental Panx1, TLR4, and SLC711A expression as well as ATF3 levels between PE cases who received antenatal steroid therapy and those who did not, implying that steroids may have no effect on the ferroptosis signaling pathway.

Panx1 channels have been linked to OS, inflammation, and cell death [48]. Panx1 isoforms oligomerize to form hexameric plasma membrane channels that are closed in physiological conditions but open in pathological ones (e.g., ischemia, hypoxia, mechanical stimulation) to release ATP and other small signaling molecules and increase membrane permeability [49]. In this study, the PE group had higher placental Panx1 mRNA expression than the normal control group. Previous studies reported that inhibiting ferroptosis production of NADPH oxidase–generated reactive oxygen species reversed cell death induced by P2Y7R, a Panx1 receptor [13, 50]. Notably, Panx1 knockdown decreased iron accumulation and iron regulatory gene expression after a ferroptosis-inducing agent, suggesting Panx1 regulates ferroptosis by reducing cellular iron [51]‏. In this study, placental Panx1 correlates positively with placental Fe^+2^ and negatively with antioxidants like placental HO-1 activity, GSH, and Gpx-4. Serum Panx1 also correlates positively with its placental expression and had a high diagnostic power of 0.997 for ferroptosis in PE.

Evidence suggests that the Panx1 channel releases ATP under OS, which combines with P2X7R to activate the mitogen-activated protein kinase (MAPK) pathway, inhibiting HO-1 expression [52], causing ferroptosis cell death. HO-1 is a cytoprotective enzyme with antioxidant activity that protects against ferroptosis [52]. A similar decrease in HO-1 activity was observed in PE [53], which was negatively correlated with Panx1 mRNA expression in this study. Taken together, our findings suggest that Panx1/HO-1 signaling contributes to ferroptosis in PE.

In a rat model of subarachnoid hemorrhage, elevated expression of Panx1 channels exacerbated the inflammatory injury associated with TLR2/TLR4/NF-κB signaling. As membrane receptors, Panx1 channels and TLR2/TLR4 can generate a “membrane to membrane” effect, facilitating ATP release from Panx1 channels to be recognized by TLRs, thereby activating the TLRs/NF-κB cascade [54]. The significant correlation of Panx1 and TLR4 with downstream signaling proteins and ferroptosis mediators in our study suggests Panx1 and TLR4 interact in ferroptosis regulation.

Although the sample size calculated for this study indicates that the samples included in this study are adequate, the small sample size remains a limitation. Further studies with larger cohorts are recommended to confirm the findings of this study.

## Conclusion

Panx1 and TLR4 may interact together to promote ferroptosis and OS in PE, offering a novel perspective on PE pathogenesis and innovative ways to diagnose and treat patients with PE.

## Data Availability

All datasets generated or analyzed during the study are included in this published article.

## Abbreviations

PE: Preeclampsia
GSH: Reduced glutathione
Gpx4: Glutathione peroxidase 4
SLC7A11: Solute carrier family 7 member 11
HO-1: Heme oxygenase-1
Panx1: Pannexin1
TLRs: Toll-like receptors
Fe^2+^: Ferrous iron
MDA: Malondialdehyde
OS: Oxidative stress
ATF3: Activating transcription factor 3
